# The role of reactive oxygen species in the transformation from prostatitis to prostate cancer: a review

**DOI:** 10.3389/fimmu.2025.1662792

**Published:** 2025-08-22

**Authors:** Kaihua Tang, Zhipeng Jiang, Wen Luo, Jing Li, DeCan Liang, Lei Liu, ZongMin Long

**Affiliations:** Department of Urology, The Third Affiliated Hospital of Zunyi Medical University (The First People’s Hospital of Zunyi), Zunyi, China

**Keywords:** reactive oxygen species, oxidative stress, prostatitis, prostate cancer, tumor microenvironment, inflammation-cancer

## Abstract

In the study of prostate diseases, the microenvironment associated with chronic prostatitis is characterized by abnormal activation of immune cells, leading to excessive accumulation of pro-inflammatory factors and an imbalance in the antioxidant defense system. This results in the overproduction of reactive oxygen species (ROS) and the subsequent triggering of oxidative stress. Oxidative stress persistently disrupts the homeostasis of prostate tissue through various mechanisms, including the damage to biomacromolecules, the regulation of inflammatory pathways, and the induction of apoptosis. ROS, as natural products of cellular metabolism, exhibit a dual role in biological systems. They are involved in the regulation of physiological signals while also possessing the potential to induce pathological damage. Further research indicates that during the occurrence and progression of prostate cancer (PCa), the gradually increasing ROS in the tumor microenvironment can activate cancer-related signaling pathways, induce Deoxyribonucleic Acid (DNA) mutations, and promote the abnormal proliferation of tumor cells. ROS are widely recognized as pivotal molecules that connect chronic inflammation to carcinogenesis. Currently, the mechanisms by which ROS mediate the cross-linking of inflammatory and carcinogenic signaling pathways during the progression from chronic prostatitis to PCa remain inadequately understood. This review systematically analyzes the multifaceted mechanisms of ROS in inflammation-induced carcinogenesis. It preliminarily elucidates the inflammatory origins of PCa and explores early intervention strategies based on the regulation of oxidative stress. The goal is to provide novel potential targets and a theoretical foundation for the comprehensive prevention and treatment of chronic prostatitis and PCa.

## 1 Introduction

Prostatitis is a prevalent urological condition affecting males across all age groups and ethnicities. The National Institutes of Health (NIH) classifies prostatitis into four distinct categories: acute bacterial prostatitis, chronic bacterial prostatitis, chronic nonbacterial prostatitis/chronic pelvic pain syndrome (CP/CPPS), and asymptomatic inflammatory prostatitis. Among these categories, CP/CPPS is the most common, representing over 90% of all prostatitis cases and significantly impacting patients’ quality of life. CP/CPPS can affect males of various age groups, with the recurrence rate progressively increasing with advancing age (1). However, the precise etiology of these conditions remains unclear and may be associated with immune dysfunction, neuroinflammatory responses, oxidative stress, endocrine disturbances, and psychological factors (2, 3). Chronic inflammation is a significant contributing factor in the initiation and progression of cancer. It modulates cellular genetic and epigenetic profiles through various molecular mechanisms, reshapes the tumor microenvironment, and creates the necessary conditions for tumor onset (4). Although a direct causal relationship between chronic inflammation and the occurrence of prostate cancer (PCa) remains unclear, some studies have indicated that prolonged chronic prostatitis may lead to persistent damage to prostate tissue, thereby creating an inflammatory environment that is conducive to carcinogenesis (5, 6).

During this process, reactive oxygen species (ROS), inflammatory mediators, and various bioactive molecules are generated through multiple signaling pathways, which synergistically target prostate tissue. Among these, ROS, characterized by its strong oxidizing properties and signal transduction capabilities, plays a crucial role in the progression from inflammation to cancer. ROS are a group of chemically reactive molecules and free radicals that contain oxygen. They primarily consist of the superoxide anion radical (O_2_·^-^), hydroxyl radical (OH·), hydrogen peroxide (H_2_O_2_), and singlet oxygen (¹O_2_) (7, 8). Under physiological conditions, ROS play a crucial role in regulating various cellular processes, including proliferation, differentiation, metabolism, apoptosis, and antioxidant defense. However, elevated intracellular ROS levels or compromised antioxidant defense mechanisms can induce oxidative stress, resulting in oxidative damage to proteins, lipids, and nucleic acids. This damage may subsequently lead to tissue injury and organ dysfunction (9–11). In PCa, ROS promote tumor proliferation, invasion, and metastasis through multiple pathways, including the regulation of the tumor microenvironment, activation of pro-cancer signaling pathways, and induction of Deoxyribonucleic Acid (DNA) damage. Therefore, further elucidation of the role of ROS in the malignant transformation from prostatitis to PCa could significantly enhance our understanding of the underlying pathogenic mechanisms involved in this progression.

This article summarizes the role of ROS in the occurrence and development of chronic prostatitis and PCa. It focuses on discussing the core role of ROS in the transformation from inflammation to cancer, aiming to elucidate how ROS facilitates the transition of chronic prostatitis into a tumor. These findings hold substantial significance for future research on the prevention and treatment of chronic prostatitis-related PCa.

## 2 The generation pathways of ROS

**Figure 1:**
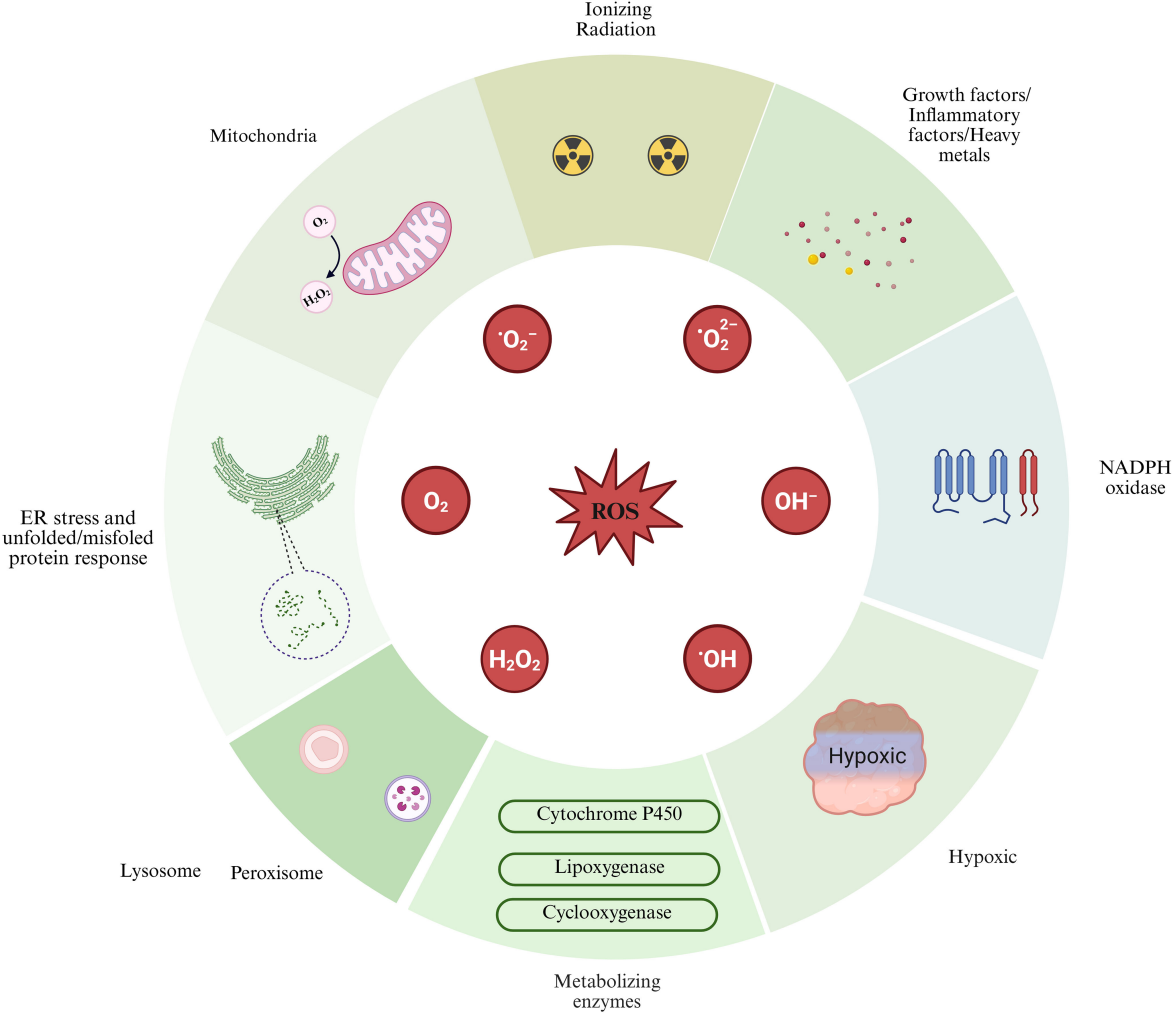
Sources of reactive oxygen species (ROS) generation are illustrated in this figure, highlighting both extracellular and intracellular contributors. Exogenous pathways include factors such as hypoxia, growth factors, inflammatory cytokines, ionizing radiation, and heavy metals. In contrast, endogenous pathways involve the mitochondria, endoplasmic reticulum (ER) stress, NADPH oxidase, lysosomes, and various cellular metabolic enzymes. Image was created with BioRender.com.

The generation pathways of intracellular ROS can be categorized into endogenous and exogenous sources. Endogenous pathways encompass the production of ROS due to mitochondrial dysfunction, endoplasmic reticulum stress, nicotinamide adenine dinucleotide phosphate (NADPH) oxidase activity, lysosomal activity, and the activation of metabolic enzyme systems, including cytochrome P450 monooxygenases, lipoxygenases, and cyclooxygenases. In the exogenous pathway, the generation of ROS is driven by extracellular stimuli, including hypoxia, growth factors, inflammatory mediators, ionizing radiation, and heavy metals (12, 13). This article primarily focuses on elucidating the critical roles of mitochondrial dysfunction, endoplasmic reticulum stress, and NADPH oxidase activation within the endogenous pathway, alongside the impact of a hypoxic environment as an exogenous stimulus in the microenvironment of the transformation from prostatitis to PCa (**Figure 1**).

### 2.1 Mitochondrial dysfunction

As the core organelle responsible for cellular energy metabolism, mitochondria inevitably experience electron leakage within their electron transport chain (ETC) during oxidative phosphorylation. Oxygen molecules react with these leaked electrons to form superoxide anions (O_2_^-^). Subsequently, under the catalytic action of superoxide dismutase (SOD), superoxide anions are converted into hydrogen peroxide. In the presence of ferrous ions (Fe²^+^), hydrogen peroxide can further generate highly reactive hydroxyl radicals through the Fenton reaction (14). ROS can induce various reactions, including protein oxidation, lipid peroxidation, and double-strand breaks in DNA. These processes may result in pathological damage and contribute to the development of diseases (15, 16). Studies have demonstrated that in PCa cell models, treatment with mannan induces significant alterations in mitochondrial structure and a pronounced decrease in mitochondrial membrane potential, indicating mitochondrial dysfunction. This is accompanied by a substantial increase in intracellular ROS levels, further corroborating that structural damage to mitochondria in PCa cells can intensify oxidative stress responses (17). Secondly, research has confirmed that in the OEx transgenic mouse model, an increase in the number of mitochondria within prostate epithelial cells, coupled with impaired mitochondrial function, leads to a significant decrease in the activity of the ETC. This results in excessive accumulation of ROS. The imbalance between the quantity and functionality of mitochondria is regarded as a critical mechanism contributing to ROS accumulation, which in turn promotes inflammatory responses and carcinogenesis (18).

### 2.2 Endoplasmic reticulum stress

During the protein folding process, the electron transfer associated with the formation of disulfide bonds can generate ROS (19). The accumulation of unfolded or misfolded proteins triggers endoplasmic reticulum stress, which exacerbates the imbalance of cellular redox, thereby intensifying ROS generation (20, 21). This creates a vicious cycle of oxidative stress and endoplasmic reticulum stress. Gara et al. have demonstrated that shikonin treatment of PCa cells significantly up-regulates the expression of glucose-regulated protein 78 (GRP78)/binding immunoglobulin protein (Bip) and C/EBP homologous protein (CHOP)/growth arrest and DNA damage-inducible protein 135 (GADD135). This effect is dependent on ROS, indicating that intracellular ROS directly participates in the regulation of the endoplasmic reticulum stress response induced by shikonin (22). Other studies have demonstrated that the natural flavonoid quercetin can elevate intracellular ROS levels in PCa treatment. It triggers endoplasmic reticulum (ER) stress responses, enhances the activation of ER stress-related signaling pathways, and promotes ROS production. Furthermore, quercetin induces cell cycle arrest at the G2/M phase and effectively inhibits tumor cell migration (23).

### 2.3 NADPH oxidase

The NADPH oxidase (Nox) family comprises seven members, namely Nox1 through Nox5, as well as Duox1 and Duox2, and plays a crucial role in the generation of ROS during inflammatory responses. This enzyme utilizes NADPH as an electron donor to facilitate the transmembrane transfer of electrons from molecular oxygen (O_2_), resulting in the production of reactive oxygen species such as superoxide anion and hydrogen peroxide (24). NADPH oxidase is predominantly expressed in phagocytes, including macrophages and neutrophils (25). Under inflammatory conditions, this enzyme generates superoxide anions through the formation of NADPH oxidase complexes. This process typically occurs on the surface of phagosomes or the cell membrane and manifests as a rapid reaction known as the respiratory burst (26). Research has shown that in the tumor microenvironment of PCa, the Nox4 subtype of NADPH oxidase is highly expressed in the stromal fibroblasts surrounding the tumor and is closely associated with the abnormal activation of transforming growth factor-β (TGF-β). Functional experiments have demonstrated that TGF-β1 can induce primary prostate fibroblasts to produce Nox4-dependent ROS, which subsequently activate the expression of cancer-associated fibroblast (CAF) markers, promoting the proliferation and migration of PCa cells. Conversely, the Nox1/4 inhibitor GKT137831 can dose-dependently inhibit ROS generation, block TGF-β1-induced fibroblast activation, matrix migration, and CAF phenotypic transformation, thus negating the tumor-promoting effect of its conditioned medium on PCa cells. Gene knockdown experiments further confirmed that the absence of Nox4 inhibits the TGF-β/SMAD signaling pathway and reduces the transcription of fibrosis-related genes, suggesting that the Nox4-ROS-TGF-β axis plays a crucial role in the stromal-epithelial interaction in PCa (27, 28). Monika Holl et al. found that the expression of the Nox5 subtype in prostate tissue is distinct. It is present in both benign and malignant prostate epithelium; however, clinical specimens of PCa exhibit a significantly higher protein level (29). The aforementioned research has elucidated the dual role of the Nox family in the onset and progression of PCa, thereby providing a theoretical foundation and empirical evidence for targeting Nox as a means to intervene in the tumor microenvironment.

### 2.4 Hypoxic environment

In hypoxic environments, the expression of hypoxia-inducible factor (HIF) is upregulated within cells, playing a crucial role in regulating the tumor microenvironment. Hypoxia can induce dysfunction of the ETC, subsequently resulting in an increase in ROS production. ROS stabilize the hypoxia-inducible factor 1-alpha (HIF-1α) protein by inhibiting the activity of prolyl hydroxylase (PHD). This inhibition activates the HIF signaling pathway and induces the expression of vascular endothelial growth factor (VEGF), thereby promoting the formation of tumor neovascularization. However, these newly formed blood vessels often exhibit structural abnormalities and functional defects, resulting in low oxygen supply efficiency. This inefficiency maintains the tumor microenvironment in a state of hypoxia, thereby creating a vicious cycle (30, 31). This mechanism promotes the formation of the tumor microenvironment and provides a theoretical basis for understanding the molecular mechanisms underlying tumor progression.

## 3 The relationship between ROS and prostatitis

### 3.1 ROS drives the inflammation of prostatitis

The level of ROS in prostate tissue exhibits a significant increase, which is closely associated with the immune response elicited by pathogen invasion or tissue damage. Upon invasion by pathogens such as bacteria and viruses, immune cells, including macrophages, are activated. These cells recognize pathogen-associated molecular patterns through pattern recognition receptors and subsequently release pro-inflammatory cytokines, including interleukin-1β (IL-1β) and tumor necrosis factor-α (TNF-α) (32). These inflammatory mediators initiate intracellular signal transduction cascades by binding to tumor necrosis factor receptor-1 (TNFR-1) and interleukin-1 receptor (IL-1R). The activation of TNF-α/IL-1β-mediated receptors can induce the phosphorylation of key proteins within the NF-κB and the mitogen-activated protein kinase (MAPK) signaling pathways, thereby triggering a cascade amplification effect. Specifically, receptor activation promotes the recruitment of adaptor proteins, activating the NADPH oxidase complex and upregulating its expression and activity, ultimately leading to excessive generation of ROS (33). A significant increase in ROS levels triggers a robust oxidative stress response. Notably, during the phagocytic process, immune cells produce substantial amounts of ROS via the respiratory burst mechanism. Although this physiological process effectively eradicates pathogens, it may also result in the establishment of a localized oxidative stress microenvironment (26, 34).

### 3.2 ROS-mediated prostate injury in preclinical studies

Zhang Yi et al. successfully established a mouse model of experimental autoimmune prostatitis (EAP) by sensitizing with prostate antigen and injecting LIT-927. This model effectively simulates the inflammatory response and the pathological processes associated with oxidative stress in chronic prostatitis. The research findings indicate that levels of lipid peroxidation products, including malondialdehyde (MDA) and 4-hydroxynonenal (4-HNE), as well as markers of DNA oxidative damage, such as 8-hydroxydeoxyguanosine (8-OHdG) and γ-H2AX (phosphorylated histone H2AX), are significantly elevated in the prostate tissue of EAP mice. This suggests that ROS-mediated oxidative damage plays a direct role in the progression of inflammation. Further *in vitro* experiments demonstrated that exogenous supplementation of α-linolenic acid effectively alleviates oxidative stress damage and inhibits apoptosis in RWPE-1 (human prostate epithelial) cells by protecting mitochondrial function and enhancing the antioxidant capacity of prostate epithelial cells (Yi 35). Feng Bin and colleagues induced autoimmune prostatitis in Sprague Dawley rats through multiple intradermal injections of a prostate protein homogenate mixed with complete Freund’s adjuvant (CFA). They also established a lipopolysaccharide (LPS)-induced human prostate epithelial cell (RWPE-1) model. Research findings indicate that in the prostate tissue of EAP rats, there is significant infiltration of inflammatory cells, accompanied by a marked increase in ROS production. This results in elevated levels of lipid peroxidation, as evidenced by increased MDA levels, and a decline in antioxidant capacity, indicated by decreased SOD and glutathione levels. These findings further confirm the crucial role of ROS in the pathogenesis of prostatitis (36). Zhao Qinxin et al. established a rat model of CP/CPPS by injecting complete Freund’s adjuvant (CFA) into the prostate. The study revealed that the CFA-induced CP/CPPS rat model exhibited significant prostate tissue damage, characterized by extensive infiltration of inflammatory cells, tissue edema, and interstitial fibrosis. Furthermore, both prostate tissue and serum analyses indicated a significant decrease in the activities of antioxidant enzymes, including catalase (CAT), total superoxide dismutase, and glutathione peroxidase (GPX) the model group, alongside a marked increase in MDA levels (37).

These studies indicate that ROS are not merely by-products of the inflammatory process; rather, they serve as crucial mediators of prostate damage. ROS play a central role in the development of prostate tissue injury by initiating oxidative stress responses.

### 3.3 Research on ROS in the clinical diagnosis of prostatitis

ROS serve as a fundamental mediator of inflammation and tissue damage in prostatitis, with related biomarkers demonstrating considerable diagnostic value. Numerous early studies have validated the role of ROS in the diagnosis of prostatitis. A.R. Shahed et al. demonstrated that, in patients with chronic prostatitis, the level of the oxidative stress marker 8-isoprostaglandin F2α (IsoP) in the ejaculatory prostatic secretion (EPS) of type II patients with Gram-positive bacterial infections is significantly higher than that in other patient types and healthy controls. Furthermore, the gene expression levels of heme oxygenase-1 (HO-1) and granzyme B are found to be elevated. Following successful treatment, IsoP levels in the EPS of patients significantly decrease, indicating that oxidative stress may represent a critical pathway and that targeted antioxidant therapy could be a promising approach (38). Pasqualotto FF et al. found that the examination of EPS revealed significantly higher levels of ROS in samples from patients with prostatitis compared to those from a healthy control group. Furthermore, the degree of granulocyte infiltration was positively correlated with ROS generation. These findings suggest that infiltrating neutrophils and macrophages may serve as the primary sources of local ROS production (39). Zhou JunF et al. conducted a dynamic monitoring study of oxidative damage indicators in patients with chronic bacterial prostatitis. Their findings indicate that as the disease progresses, levels of oxidative products, such as nitric oxide in plasma and MDA in red blood cells, exhibit a progressive increase. Concurrently, the activities of SOD, CAT, and GPX in red blood cells show a gradual decline. These results confirm the pathological characteristic of a continuous aggravation of oxidative stress during the chronic phase of inflammation (40). Jian-Guo Lou et al. conducted a case-control study that revealed significantly higher average levels of MDA and SOD in patients with chronic bacterial prostatitis compared to the healthy control group (41).

In clinical diagnosis, the detection of ROS and their associated markers is crucial for the early identification of prostatitis, providing a foundation for disease assessment and therapeutic monitoring. An elevated level of MDA in serum or EPS indicates significant lipid peroxidation and serves as an indirect indicator of tissue damage. The observed decrease in the activities of SOD, CAT, and GPX indicates a diminished capacity of the body to eliminate free radicals, which may suggest the ongoing progression of inflammation. Consequently, integrating these biomarkers into a comprehensive diagnostic system for prostatitis could enhance diagnostic accuracy and sensitivity, thereby providing a scientific foundation for the development of personalized treatment plans.

## 4 The relationship between ROS and PCa

The occurrence and development of PCa are closely associated with the remodeling of cellular metabolism and the imbalance of redox homeostasis. ROS, as signaling molecules with potent oxidizing and signaling functions, can induce oxidative damage to DNA, proteins, and lipids when present at excessive levels. Redox imbalance is not only an inevitable consequence of tumor metabolic reprogramming but also one of the core mechanisms driving the malignant progression of PCa.

### 4.1 ROS generation in PCa

Tumor cells establish a unique energy metabolic network through metabolic reprogramming, with the most notable feature being the aberrant activation of aerobic glycolysis, commonly known as the Warburg effect (42). The Warburg effect has consistently been demonstrated as a significant metabolic characteristic of advanced PCa cells (43). Enhanced glycolysis can indirectly elevate NADPH levels by supplying substrates for the pentose phosphate pathway, which may further contribute to the generation of ROS (44). Xia et al. found that ROS in PCa cells may originate from NADPH oxidase, specifically the Nox4 subtype, and the mitochondrial respiratory chain, as both the NADPH oxidase inhibitor diphenyleneiodonium (DPI) and the mitochondrial complex I inhibitor rotenone significantly inhibited ROS generation (45). Studies have demonstrated that subtypes such as Nox4 and Nox5 are highly expressed in PCa tissues. The abnormal upregulation of their activity can significantly enhance the production of ROS (29, 46). Secondly, mitochondrial dysfunction plays a crucial role in the molecular mechanisms underlying the imbalance of ROS generation (47). In conditions characterized by disrupted electron transfer homeostasis within the mitochondrial electron transport chain, superoxide anion (O_2_^-^) is produced, representing an abnormal mechanism for the generation of mitochondrial reactive oxygen species (mtROS) (48). The abnormal production of mtROS has significant pathophysiological implications. Studies have demonstrated that the accumulation of mtROS in PCa is significantly positively correlated with the invasive and migratory capabilities of tumor cells (49, 50).

### 4.2 The pro-proliferative and anti-apoptotic effects of ROS on tumor cells

The pro-proliferative and anti-apoptotic effects of ROS on tumor cells are of great significance. As a signaling molecule, ROS activates multiple intracellular signaling pathways that are closely related to cellular proliferation. For instance, the MAPK signaling pathway, which includes extracellular signal-regulated kinase (ERK), c-Jun N-terminal kinase (JNK), and p38 MAPK (51). In PCa cells, an increase in intracellular ROS levels can activate the ERK signaling pathway. Phosphorylated ERK translocates to the nucleus, where it regulates the expression of a series of genes associated with the cell cycle process, thereby facilitating the transition of cells from the G1 phase to the S phase and significantly enhancing cell proliferation (52–54). Research has demonstrated that ROS can activate the JNK signaling pathway and the DNA damage response, which may induce cancer cells to enter a state of growth arrest or senescence, ultimately leading to apoptosis. The activation of JNK subsequently activates the AP-1 transcription factor, thereby initiating a cascade of downstream pathways closely associated with apoptosis, including p53 and PARP-1, and facilitating the apoptotic process (55). In the context of apoptosis regulation, ROS can influence both the expression and activity of apoptosis-related proteins. An increase in ROS production results in an upregulation of the ratio between Bcl-2 associated X protein (Bax) and B-cell lymphoma 2 (Bcl-2), thereby promoting apoptosis (56). Bcl-2 family proteins are pivotal in the mitochondrial-mediated apoptotic pathway (57).

Studies have demonstrated that sesquiterpenes can induce apoptosis by inhibiting the Phosphatidylinositol 3-Kinase (PI3K)/Protein Kinase B (AKT)/Mammalian Target of Rapamycin (mTOR) signaling pathway. This inhibition reduces mitochondrial membrane potential and promotes the release of cytochrome c, which subsequently activates the caspase cascade reaction. Furthermore, this process is associated with an increase in ROS production, down-regulation of anti-apoptotic proteins such as Bcl-2, and up-regulation of pro-apoptotic proteins including p53 and p21 (58). This process indicates that enhancing the stability of the mitochondrial membrane and preventing the release of cytochrome C from mitochondria into the cytoplasm can further inhibit the occurrence of the apoptotic cascade reaction. Meanwhile, ROS can regulate the intracellular redox state and influence the activity of key proteins within the apoptotic signaling pathway. The caspase family, which serves as the primary executor proteins in apoptosis, has its activity strictly regulated by the intracellular redox state. ROS can inactivate caspases by oxidizing cysteine residues, ultimately achieving an inhibitory effect on apoptosis. (59).

The aforementioned research indicates that ROS play a dual regulatory role in PCa. On one hand, ROS can promote cell proliferation by activating signaling pathways such as the MAPK pathway; on the other hand, they exert anti-apoptotic effects by modulating the mitochondrial apoptotic pathway and influencing caspase activity. This dual functionality is crucial for the survival of PCa cells and contributes to their clonal advantage. Furthermore, the imbalance in the bidirectional regulatory mechanisms of ROS is considered a significant driving factor in the malignant progression of tumors.

## 5 The influence of ROS on the progression from prostatitis to PCa

### 5.1 ROS damage the tissue and cellular structure of the prostate

Under chronic inflammatory conditions, the intracellular environment undergoes significant changes, characterized by a sharp increase in ROS levels. Prolonged stimulation by inflammatory factors maintains the generation of ROS in an active state, thereby creating a robust microenvironment of oxidative stress. This environment can lead to cellular dysfunction and may even induce apoptosis or necrosis by promoting lipid peroxidation of cell membranes. This process disrupts the integrity of cellular structures and results in mitochondrial dysfunction, thereby exacerbating the pathological damage to prostate tissue (60).

ROS can interact with polyunsaturated fatty acids (PUFAs) within the phospholipid bilayer of cell membranes, thereby initiating a chain reaction of lipid peroxidation that disrupts the integrity and permeability of the cell membrane. Among these, the hydroxyl radical (·OH) exerts the most significant impact on lipids. Hydroxyl radicals attack polyunsaturated fatty acids in cell membranes, generating toxic aldehyde products such as MDA and 4-hydroxynonenal (4-HNE). These products lead to decreased membrane fluidity, increased permeability, and ion gradient imbalance. Furthermore, they are frequently utilized as biomarkers for assessing the extent of oxidative damage (61). In prostate tissue, lipid peroxidation may compromise the barrier function of epithelial cells, increase tissue permeability, promote the infiltration of inflammatory cells, and exacerbate local inflammatory responses. These pathological changes can further intensify tissue damage and stimulate enhanced proliferation of epithelial cells to repair the damaged tissue, potentially inducing the occurrence of PCa (62, 63). Abnormal remodeling of lipid metabolism is a fundamental biological characteristic of cancer, significantly influencing the occurrence, development, and metastasis of tumors. Biesiadecki et al. conducted a study involving 50 PCa patients and found that the MDA level in these patients was higher than that in healthy men, while the total antioxidant capacity in urine was significantly reduced. The elevated MDA level, a byproduct of lipid peroxidation, suggests that oxidative stress-induced tissue damage plays a crucial role in the onset and progression of PCa (64). Meltem Ozlen Dillioglugil et al. found that plasma MDA levels in PCa patients were significantly higher than those in healthy controls (65). This increase in lipid peroxidation products in PCa suggests their potential role in the etiology or progression of the disease.

Secondly, mitochondria serve as primary targets for ROS (66). ROS can damage key components of the inner mitochondrial membrane, notably cardiolipin. As a phospholipid exclusive to the inner mitochondrial membrane, cardiolipin is essential for maintaining the structural and functional integrity of electron transport chain complexes, including complexes I, III, and IV. ROS modifies cardiolipin through oxidation, disrupting its normal interactions with these complexes. This modification leads to decreased stability of the complexes, reduced electron transfer efficiency, and increased electron leakage. Ultimately, this exacerbates ROS generation, creating a self-reinforcing vicious cycle (66, 67). The reduction in Adenosine Triphosphate (ATP) synthesis, resulting from the disruption of electron transport chain function, leads to disorders in cellular energy metabolism, thereby affecting the normal physiological functions of cells (68). This insufficiency further compromises the energy supply for prostate epithelial and stromal cells, exacerbating tissue damage and inflammatory responses (69).

In chronic prostatitis, ROS induce lipid peroxidation of cell membranes, disrupt the barrier function of cells, and damage mitochondria, leading to energy metabolism disorders. This process results in oxidative stress damage within prostate tissue, intensifies the inflammatory microenvironment, and promotes abnormal cell proliferation through a dual mechanism. Consequently, this lays a structural and functional pathological foundation for the occurrence and progression of PCa.

### 5.2 The chronic accumulation of ROS creates a microenvironment conducive to the formation of PCa

Chronic inflammation is a significant factor influencing the occurrence and progression of PCa to advanced metastatic disease. The use of anti-inflammatory drugs and antioxidants has been shown to reduce the risk of developing PCa (70). Under chronic inflammatory conditions, the likelihood of cellular mutations and genetic alterations increases, which may facilitate the occurrence and progression of tumors. The persistent activation of various inflammatory signaling pathways, growth factors, and cellular messengers maintains pro-inflammatory genes and oncogenes at elevated expression levels for extended periods, thereby heightening the risk of genomic instability and gene mutations. This ultimately fosters a microenvironment that is conducive to carcinogenesis (71).

The tumor microenvironment (TME) is a dynamic and complex multicellular environment composed of various cell types, including tumor cells, blood vessels, fibroblasts, immune cells, as well as vascular and lymphatic networks and the extracellular matrix (72, 73). The TME is a critical factor in tumor progression. Within the TME, the continuous accumulation of ROS produced by mitochondrial and other pathways can exacerbate cancer deterioration.

In the tumor microenvironment, macrophage phenotypic polarization exhibits significant functional differences, primarily classified into the M1 phenotype, which has tumor-suppressive effects, and the M2 phenotype, which promotes tumor development (74). The activation of the M1 phenotype is closely associated with increased ROS production, elevated interleukin-12 (IL-12) expression, and decreased interleukin-10 (IL-10) expression (75). This phenotype produces pro-inflammatory mediators such as IL-6, IL-1β, IL-12, and TNF-α (76, 77).

Research has confirmed that ROS can promote the polarization of macrophages in the TME toward the M1 phenotype (78). In contrast, the M2 phenotype, characterized by the expression of cytokines such as interleukin-4 (IL-4), interleukin-13 (IL-13), and IL-10, exhibits tumor-promoting and angiogenic functions (79). Tumor-associated macrophages (TAMs) typically demonstrate M2 polarization (80). ROS plays a crucial regulatory role in macrophage polarization, with its production during macrophage differentiation primarily controlled by Nox isoenzymes. For example, ROS generated by Nox1 and Nox2 can induce the differentiation of mouse monocytes into macrophages, thereby enhancing their polarization toward the M2 phenotype and TAM (81).

At the level of immune cells, ROS produced by macrophages exert a potent toxic effect on natural killer (NK) cells and T cells, inducing their apoptosis (82). The activation, proliferation, and functional maintenance of T cells are dependent on low levels of ROS. However, within the TME, elevated ROS levels hinder T cell proliferation and anti-tumor functionality, thereby diminishing their reactivity (83). The release of ROS by T regulatory cells and TAMs significantly impairs the cytotoxic responses of both NK cells and T cells (84). ROS can induce apoptosis in T cells. Elevated levels of ROS in T cells and NK cells can lead to the down-regulation of T cell receptor ζ chain and CD16ζ chain expression. This down-regulation inhibits the activation of the NF-κB signaling pathway, resulting in insufficient production of cytokines such as interferon-gamma (IFN-γ), TNF-α, and interleukin-2 (IL-2) (85, 86). Hypoxia within the TME is a critical factor that significantly diminishes the expression of activating receptors on NK cells. In response to hypoxic conditions, NK cells adapt by upregulating HIF-1α; however, this adaptation concurrently inhibits their capacity to enhance the surface expression of key activating receptors, including NKp46, NKp30, NKp44, and NKG2D (87, 88).

From the perspective of metabolic regulatory mechanisms, the Warburg effect represents a predominant metabolic characteristic of advanced PCa cells. Notably, even in the presence of oxygen and fully functional mitochondria, there is a marked increase in the rate of glucose uptake, which subsequently leads to elevated lactic acid production (89–91). Moreover, the Warburg effect generates ROS by altering the mitochondrial redox potential (92). ROS can induce oxidative stress in cancer-associated fibroblasts, promoting their transition to glycolytic metabolism, which produces high levels of lactate for ATP production. This process establishes a metabolic synergy network that meets the energy demands of the tumor microenvironment. Such metabolic reprogramming is especially pronounced in metastatic castration-resistant PCa (93).

ROS induced by chronic prostatitis contribute to the establishment of a pro-cancer microenvironment within the tumor microenvironment through mechanisms such as immunosuppression and metabolic remodeling. Their persistent accumulation serves as a link between inflammation and carcinogenesis, thereby facilitating the onset and progression of PCa.

### 5.3 Aberrant activation of the ROS signaling pathway promotes the occurrence and development of PCa

As a redox messenger, ROS play a significant role in the progression of PCa by regulating various signaling molecules, including transcription factors such as PI3K, HIFα, and NF-κB. These factors are sensitive to the redox state and can modulate downstream signaling pathways.

An increase in ROS levels in PCa can lead to the dysregulation of the PI3K signaling pathway. This dysregulation occurs through the inhibition of phosphatase activity, which promotes the enhancement of AKT signaling, thereby facilitating cell proliferation and survival (94). The abnormal activation of the PI3K-AKT pathway may result in increased cell invasiveness and promote the progression of PCa (95). Lu Jin et al. found that treatment of PCa cells with the dual PI3K/mTOR inhibitor GNE-493 resulted in a significant increase in ROS levels. Concurrently, a marked enhancement of lipid peroxidation and an increase in DNA damage were observed (96). Hypoxia, a characteristic feature of the tumor microenvironment, promotes the production of ROS by mitochondria, which stabilizes the HIFα subunit and activates the HIF signaling pathway (94). HIF-1α facilitates the adaptation of tumor cells to hypoxic environments by activating the transcription of relevant genes. It is closely associated with the expression of factors such as VEGF, thus participating in the angiogenesis process of PCa and influencing the tumor’s malignancy and progression (97, 98). In PCa, NF-κB is persistently activated and plays a crucial role in promoting angiogenesis, anti-apoptotic mechanisms, and the production of metastasis-promoting factors, thereby exerting a carcinogenic effect (99, 100). Research has shown that inhibiting NF-κB activity in the signaling pathway of DU145 PCa cells can induce the activation of JNK kinase, which subsequently leads to the phosphorylation of serine 15 on the p53 tumor suppressor gene. This process enhances the stability, DNA binding ability, and pro-apoptotic function of p53 (101, 102).

ROS can promote the abnormal proliferation and malignant transformation of tumor cells through various pro-cancer signaling pathways. The dysregulation of the signaling network mediated by ROS is regarded as a crucial molecular mechanism that connects chronic inflammation to tumorigenesis. In cases of acute pancreatitis, mitochondrial dysfunction can result in the excessive production of ROS, which in turn triggers oxidative stress. This oxidative stress damages cellular membranes, nucleic acids, and proteins, ultimately leading to the apoptosis and necrosis of pancreatic cells (103). Studies have demonstrated that ROS play a crucial role in cell transformation induced by the mutant oncogenic KRAS and the proliferation of pancreatic tumors. Oxidative stress interacts with mutant KRAS, collaboratively promoting the initiation and progression of pancreatic cancer. Specifically, mutant KRAS elevates ROS levels derived from mitochondria, which in turn induces acinar cells to undergo metaplasia into ductal epithelium, ultimately leading to the development of pancreatic intraepithelial neoplasia (104). During the occurrence and progression of pancreatic ductal adenocarcinoma, ROS can facilitate the malignant transformation of cells and expedite tumor development by activating signaling pathways such as NF-κB (105). In cholangitis, prolonged and persistent inflammatory stimulation induces inflammatory cells, such as Kupffer cells, to secrete a variety of mediators, thereby establishing a tumor-promoting microenvironment. During the malignant transformation of bile duct cells, ROS contribute to the occurrence and progression of cancer through multiple mechanisms, including the induction of oxidative damage, activation of relevant signaling pathways—such as the JNK pathway that mediates cell proliferation and transformation—and regulation of the TME (106). Detian Yuan et al. have demonstrated that liver injury mediated by Kupffer cells can promote the proliferation and carcinogenic transformation of bile duct cells by inducing the generation of ROS and the paracrine effect of TNF, thereby activating the JNK signaling pathway. However, intervention measures such as antioxidant therapy, depletion of Kupffer cells, knockout of the Tnfr1 gene, or inhibition of JNK activity can effectively reduce the incidence of precancerous lesions in bile duct cells (107). During this process, ROS facilitate the malignant transformation of bile duct cells by inducing oxidative damage and activating multiple signaling pathways. These mechanisms suggest that the role of ROS in promoting tumorigenesis may exhibit cross-organ universality, providing a significant reference for a deeper understanding of its involvement in the transformation from prostatic inflammation to cancer.

### 5.4 Chronic accumulation of ROS leads to gene mutations

The occurrence of tumors is closely linked to genetic alterations and progressive phenotypic abnormalities. In PCa, elevated levels of ROS can induce oxidative damage to biomolecules, including DNA, lipids, and proteins. This damage leads to gene mutations and initiates early events associated with tumor initiation and progression.

ROS-mediated DNA damage plays a crucial role in the initiation and malignant transformation associated with carcinogenesis. ROS can induce DNA damage by cleaving the phosphodiester bonds in DNA, which leads to single-strand or double-strand breaks. Additionally, ROS can oxidize and modify the pyrimidine and purine bases, resulting in structural alterations (108). ROS directly target both nuclear DNA and mitochondrial DNA (mtDNA), resulting in a dual damage effect. At the level of the nuclear genome, ROS can induce the oxidation of guanine bases, leading to the formation of 8-OHdG. This compound is one of the primary forms of oxidative damage caused by ROS and is widely recognized as a biomarker for oxidative stress and carcinogenesis (109, 110). Shinji Ohtake et al. found that the expression of 8-OHdG in PCa tissues is significantly higher than that in benign prostate tissues (111). The accumulation of 8-OHdG can lead to genetic damage due to base mismatches (112). At the mitochondrial level, ROS can easily induce oxidative damage to mtDNA, resulting in mtDNA mutations and the activation of the cGAS-STING innate immune pathway (113). Activated STING can directly stimulate downstream IκB kinase (IKK), leading to the release of NF-κB (114). Furthermore, NF-κB signaling can also be activated by STING (115). Clinical research has confirmed that the levels of 8-OHdG in the prostate tissue of patients with prostatitis are significantly elevated, indicating the presence of DNA damage mediated by oxidative stress (116). If this damage is not repaired promptly, it may induce abnormal proliferation of tumor cells through continuous genomic instability, ultimately leading to malignant transformation (117).

**Figure 2:**
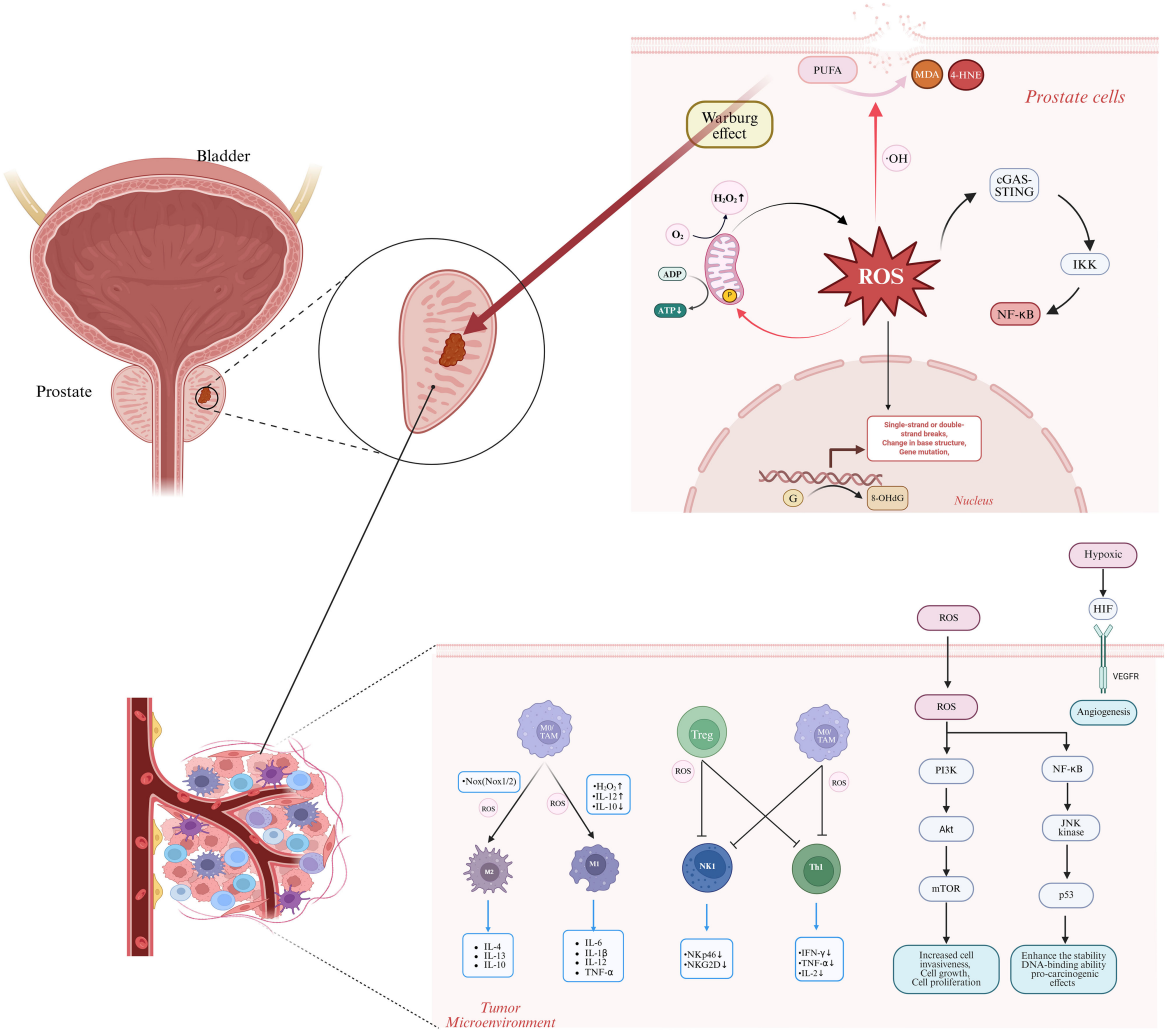
The role of reactive oxygen species (ROS) in the progression from prostatitis to prostate cancer encompasses several critical mechanisms. ROS interact with polyunsaturated fatty acids (PUFAs) to generate toxic aldehydes, such as malondialdehyde (MDA) and 4-hydroxynonenal (4-HNE), resulting in cell membrane disruption. ROS target cardiolipin in the inner mitochondrial membrane, which exacerbates ROS production and impairs ATP synthesis. ROS dysregulate immune cells by promoting M1 macrophage polarization, thereby increasing pro-inflammatory mediators, and also drive M2 macrophage activation, leading to the generation of tumor-promoting cytokines. ROS suppress T cell and natural killer (NK) cell functions by downregulating activation receptors, including NKp46 and NKG2D. In terms of oncogenic signaling pathways, ROS activate PI3K-AKT, NF-κB, HIF, and other pathways, promoting prostate cancer cell proliferation, survival, and malignant transformation. ROS induce genetic damage through guanine oxidation, as indicated by the presence of 8-OHdG, and enhance the Warburg effect, which provides energy for tumor cell proliferation. ROS activate the cGAS-STING pathway, triggering IKK activation and subsequent NF-κB release. OH, hydroxyl radical; PUFA, polyunsaturated fatty acid; MDA, malondialdehyde; 4-HNE, 4-hydroxynonenal; 8-OhdG, 8-hydroxydeoxyguanosine; NF-κB, nuclear factor κB; TAM, tumor-associated macrophage; Treg, regulatory T cell; NKp46, natural killer cell activation receptor protein 46; NKG2D, natural killer group 2 member D; IFN-γ, interferon-γ; JNK, c-Jun N-terminal kinase; HIF, hypoxia-inducible factor; PI3K, phosphatidylinositol 3-kinase; AKt, protein kinase B; mTOR, mammalian target of rapamycin. Image was created with BioRender.com.

ROS-mediated gene mutations serve as a significant molecular initiating factor for the development of PCa. These mutations primarily exert their effects through direct damage to the nuclear genome and the disruption of signaling pathways resulting from mitochondrial dysfunction. This synergistic pathogenic mechanism, involving multiple targets and pathways, provides a crucial theoretical foundation for antioxidant treatments and targeted intervention strategies in PCa (**Figure 2**).

## 6 Antioxidants are key substances in the treatment of prostatitis and the prevention of PCa

Antioxidants play a significant role in the treatment of prostatitis and the prevention of PCa due to their multifaceted mechanisms. Their primary mechanisms of action include reducing oxidative stress, inhibiting cancer cell proliferation, and enhancing treatment sensitivity, among others. Specifically, various antioxidants such as vitamin D, selenium, zinc, lycopene, resveratrol, curcumin, and quercetin exhibit unique benefits in these two types of diseases through distinct action pathways. Vitamin D3 has been shown to improve experimental prostatitis, and serum vitamin D levels exhibit a negative correlation with the risk of PCa. Additionally, selenium not only alleviates chronic prostatitis but also reduces the incidence of PCa. The effects and research findings regarding various antioxidants on prostatitis and PCa are summarized in **Table 1**. These research findings provide theoretical basis and potential intervention targets for optimizing the treatment strategies of prostatitis and innovating the preventive measures of PCa.

**Table 1:** Effects or research findings of antioxidants on prostatitis and PCa.

Serial No.	Antioxidant	Effects on Prostatitis	Effects on PCa	References
1	Curcumin	Curcumin suppresses the expression of inflammatory factors and MDA activity in rat models of CP/CPPS.	It reduces the proliferation of PCa cells and enhances the activity of programmed cell death.	(118, 119)
2	Lycopene	Lycopene has been shown to effectively alleviate CP/CPPS by reducing inflammatory responses and oxidative stress.	A high intake of lycopene is inversely correlated with a reduced risk of PCa.	(37, 120, 121)
3	Quercetin	Quercetin has been shown to prevent chronic prostatitis in rat models by modulating the NF-κB and MAPK signaling pathways.	It effectively downregulates cell survival, proliferation, and anti-apoptotic proteins in PCa cells.	(122, 123)
4	Resveratrol	Resveratrol activates Sirt1 to downregulate c-kit/SCF, thereby mitigating the progression of chronic prostatitis.	It exerts anti-cancer effects on PCa cells, either alone or in combination with ionizing radiation, and can delay or prevent the onset of PCa.	(124–126)
5	Selenium	Selenium demonstrates protective effects against chronic non-bacterial prostatitis induced by 17β-estradiol.	Through its antioxidant and immunomodulatory properties, selenium also decreases the incidence of PCa.	(127, 128)
6	Vitamin D	VVitamin D3 enhances macrophage polarization during EriB treatment, thereby improving the symptoms of experimental autoimmune prostatitis.	Low serum levels of vitamin D are correlated with an increased risk of PCa.	(129, 130)
7	Zinc	Intraprostatic zinc injection has been shown to inhibit bacterial growth and may provide therapeutic benefits in the management of patients with NIH-IIIA chronic prostatitis.	Reduced zinc levels are associated with decreased apoptosis of malignant cells and an increase in the proliferation of PCa cells.	(131–133)

EriB, Eriocalyxin B; NIH-IIIA, National Institutes of Health–IIIA; Sirt1, Silent Information Regulator 2 Homolog 1; c-kit/SCF, Cell Surface Tyrosine Kinase Receptor/Stem Cell Factor; MDA, Malondialdehyde; NF-κB, Nuclear Factor kappa-B; MAPK, Mitogen-Activated Protein Kinases.

Despite the progress made in current research, numerous challenges and controversies remain. For instance, the Selenium and Vitamin E Cancer Prevention Trial (SELECT) demonstrated that supplementation with either selenium or vitamin E did not reduce the risk of PCa and may have even increased it. Notably, vitamin E supplementation was found to significantly elevate the risk of PCa in healthy men (134). This contradictory conclusion indicates that the clinical application of antioxidants may involve a complex dose-effect relationship and individual variability. The human body’s antioxidant system is an interconnected network formed by the coordinated action of multiple components. Its functionality depends on the synergistic effects of all components rather than the action of a single molecule. Excessive or inappropriate supplementation of antioxidants may disrupt the body’s inherent balance, potentially leading to adverse health effects (135, 136). Physiological doses of antioxidants may offer potential benefits for treating prostatitis and preventing PCa. However, exogenous supplementation exceeding physiological needs may yield adverse effects. A meta-analysis suggests that neither vitamin E supplements nor high dietary intake of vitamin E is likely to significantly influence the chemoprevention of PCa (137). Current clinical studies are limited in reliability due to insufficient sample sizes, short follow-up periods, and inadequate control of confounding factors. Therefore, future research should focus on conducting multi-center, large-sample, and long-term follow-up clinical studies to systematically verify the effectiveness and safety of antioxidants. Additionally, a precise antioxidant intervention plan should be formulated based on the individual genetic and metabolic characteristics of the patient, as well as the stage of the disease. Finally, it is essential to strengthen the connection between basic research and clinical application, clarifying the synergistic effects of antioxidants with other treatment methods, and optimizing prevention and treatment strategies for prostatitis and PCa.

## 7 Future perspectives

### 7.1 Clinical translation

The application of ROS in the clinical translation of prostate diseases illustrates that changes in the levels of related oxidative stress biomarkers can serve as effective diagnostic indicators and tools for assessing disease progression in prostatitis and PCa. Intervention strategies that focus on ROS regulation present a promising approach for the prevention and treatment of prostatitis and PCa, thus holding considerable significance in advancing the translation of fundamental research findings into clinical applications.

Oxidative stress and its associated biomarkers play a critical role in the pathophysiological progression of chronic prostatitis and PCa, with accumulating evidence elucidating their essential functions. However, challenges persist in their application for clinical differential diagnosis. Previous studies have confirmed that ROS-mediated oxidative stress significantly contributes to chronic prostatitis. Furthermore, oxidative stress markers have been detected in the genital secretions and urine of affected patients (39, 138, 139). Kaba et al. reported that MDA levels in PCa patients were significantly elevated compared to those in the control group, while the levels of SOD, GPX, and CAT were markedly reduced (140). Byeongsang Oh et al. further confirmed through a systematic review of case-control studies that the levels of oxidative stress markers in PCa patients exhibited a significant upward trend (141). Furthermore, Biesiadecki et al. observed that the concentrations of lipid peroxidation products, such as MDA, progressively increase during the progression of PCa, indicating a close association between oxidative stress levels and disease progression (142). Currently, the absence of specific diagnostic thresholds makes it challenging to accurately distinguish whether patients are in the stages of simple prostatitis, precancerous lesions, or early PCa.

In the field of preventing and treating prostatitis and PCa, therapeutic strategies that regulate ROS have shown significant potential for application. Notably, several minimally invasive treatment methods have garnered considerable attention due to their advantages in precise targeting. With advancements in technology, drug delivery systems utilizing nanoparticles (NPs) have become increasingly prevalent (143). The application of nanoparticles in the treatment of inflammation, as well as in radiotherapy and chemotherapy for cancer, offers novel approaches for addressing prostatitis and PCa. Nanoparticles developed by Bingliang Chen et al. have demonstrated significant clinical potential in patients with chronic non-bacterial prostatitis, exhibiting strong anti-inflammatory effects, specifically by reducing oxidative stress levels and enhancing antioxidant responses (89). Xiao-Xiao Guo et al. developed a nanoparticle-based radiosensitization strategy for PCa that integrates dose deposition enhancement, induction of cell cycle arrest, and ROS generation, demonstrating promising therapeutic outcomes (144). Zou et al. developed a novel LET-SeNP nanocarrier system that enhances the efficacy of radiotherapy in PCa by downregulating thioredoxin reductase expression, inducing excessive ROS production, and subsequently activating the mitochondrial-mediated apoptotic signaling pathway (145). In addition, photodynamic therapy (PDT) can stimulate anti-tumor immune responses, disrupt the tumor microvascular system, and induce apoptosis or cell death in tumor cells. This is achieved by combining specific wavelengths of light with photosensitizers to produce ROS (146). Clinical research has confirmed the effectiveness of vascular-targeted photodynamic (VTP) therapy (147). A Phase II clinical trial conducted in France demonstrated its efficacy. Furthermore, a follow-up study in Germany has indicated that VTP therapy is a promising treatment option for patients with unilateral low-risk PCa (148). Additionally, sonodynamic therapy (SDT) operates through a ROS-mediated regulatory mechanism. This approach combines low-intensity ultrasound with sonosensitizers to induce the production of cytotoxic ROS, thereby facilitating targeted ablation of tumor sites and direct destruction of PCa cells (149). Maryam Mohammad Hadi et al. have developed a novel nanoparticle-based delivery system for the targeted treatment of PCa using SDT, which shows promising potential in terms of both safety and therapeutic efficacy (150).

In the future, multicenter clinical cohorts encompassing various stages of prostate diseases should be established to facilitate a comprehensive investigation of ROS-associated signaling pathways. This will help define the clinical thresholds of oxidative stress biomarkers, identify combinations of diagnostic markers, and integrate these with traditional biomarkers such as PSA, thereby enhancing the capacity to detect prostatitis and early-stage PCa. Ongoing research into the regulatory mechanisms of ROS, along with the continuous refinement of therapeutic techniques and the development of novel delivery systems, is anticipated to enhance the targeting precision and safety profile of PCa treatment. Moreover, the potential applications of these advancements in the prevention and management of prostatitis merit close attention, as they may significantly contribute to the progression of precision medicine for both prostatitis and PCa. These strategies could play a pivotal role in integrated treatment approaches, thereby fostering innovation in therapeutic paradigms within this field.

### 7.2 Frontier methodologies

The continuous advancement of frontier methodologies has provided innovative research tools that facilitate in-depth investigations into the mechanisms of ROS in prostatitis and PCa, including the application of organoid models and spatially resolved transcriptomic technologies.

Organoid models are three-dimensional *in vitro* culture systems derived from primary cells. They effectively replicate the tissue architecture, cellular heterogeneity, and microenvironmental characteristics of *in vivo* organs. This model has been preliminarily utilized in PCa research, yielding significant findings (151, 152). Mirjam Blattner et al. elucidated the functional role of SPOP mutations within the PI3K/mTOR pathway and androgen receptor signaling by utilizing mouse prostate organoid models and human PCa samples (153). The ROS-mediated oxidative stress process is intricately linked to the maintenance of cellular microenvironmental homeostasis and the regulation of organ function. Consequently, organoid models can be utilized to simulate *in vivo* physiological conditions, thereby facilitating the elucidation of underlying molecular mechanisms. Several studies have produced significant findings. Myeong Joon Lee et al. demonstrated, through the establishment of a prostate organoid model, that kaempferol mitigates tissue damage by inhibiting mitochondrial ROS production (154). Xiang Chen et al. examined the therapeutic effects of resveratrol on organoid models derived from bladder cancer patients, revealing that its anti-tumor activity is closely associated with multiple oxidative stress-related mechanisms, specifically including mitochondrial dysfunction and lipid peroxidation (89, 155). Moreover, spatially resolved transcriptome technology facilitates the precise localization and spatial distribution analysis of mRNA molecules in tumor tissue studies, and has become a widely adopted approach for characterizing the molecular landscape of PCa. Taghreed Hirz et al. systematically characterized the TME by integrating single-cell RNA sequencing with spatially resolved transcriptomic technologies. This approach revealed tumor-dependent alterations that differ significantly from those observed in healthy tissues (156). Junyi Hu et al. proposed that two specific luminal cell types may jointly serve as the origin of PCa based on a spatially resolved transcriptomic analysis (157). Martin Smelik et al. successfully identified PCa biomarkers that demonstrate stable expression across various biological environments and can be measured using conventional detection techniques. This was achieved through the integration of spatially resolved transcriptomic technology, pseudo-time analysis, and machine learning approaches, thereby establishing a theoretical foundation for the development of potential early diagnostic strategies (141).

**Figure 3:**
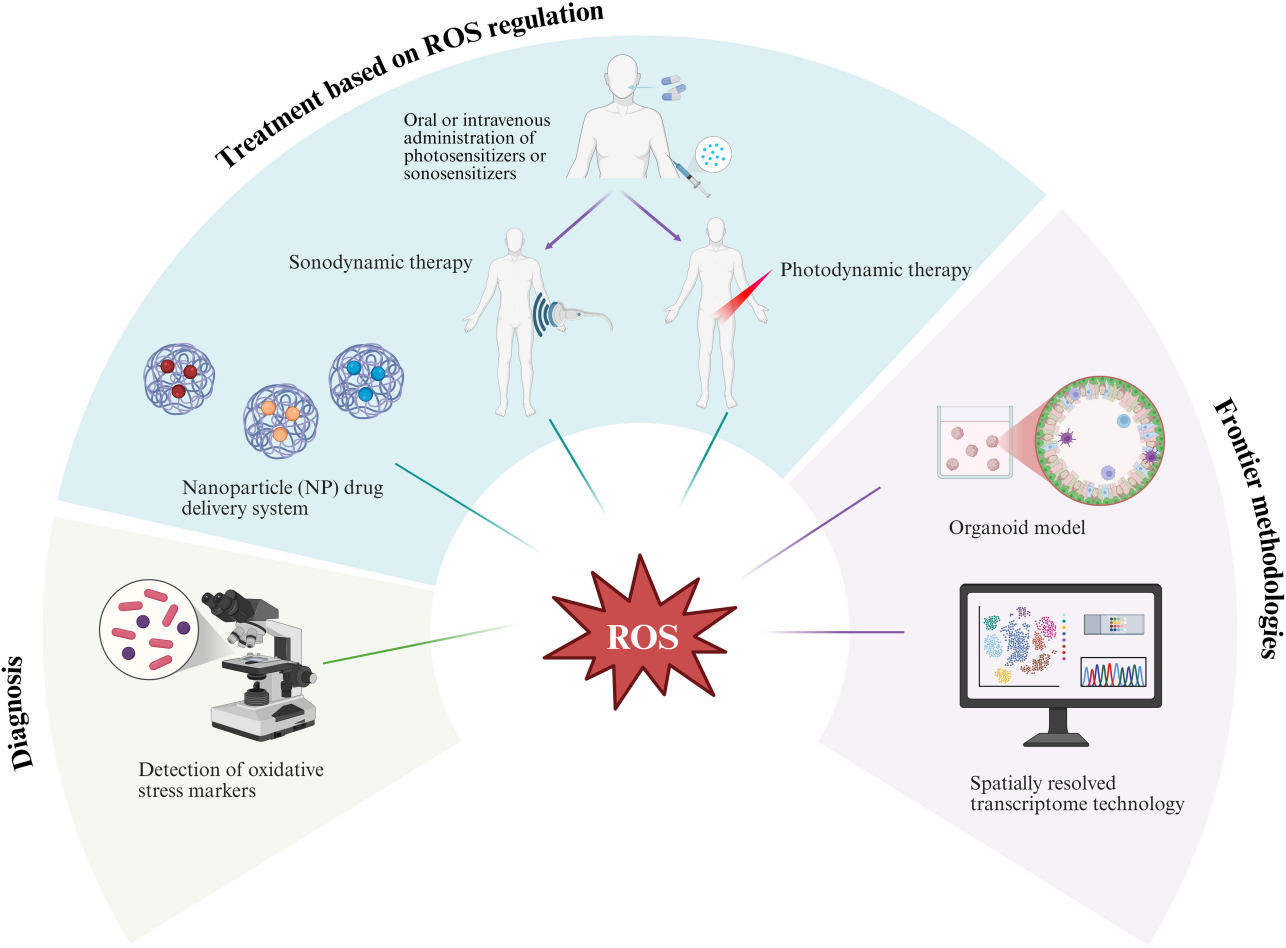
The prospects of reactive oxygen species (ROS) in the study of prostatic inflammation and its transformation into prostate cancer are significant. This research involves screening potential oxidative stress markers of value and integrating organoid models with spatially resolved transcriptome technology to thoroughly investigate the underlying mechanisms. Furthermore, it explores the combined application of innovative treatment methods, including nanoparticle drug delivery systems, photodynamic therapy, and sonodynamic therapy. Image was created with BioRender.com.

Looking ahead, the integration of key cellular components, such as mesenchymal and immune cells, into organoid models is essential for enhancing their biological fidelity and more accurately recapitulating the complexity of *in vivo* tissues (158). Thus, it can more accurately simulate the dynamic process of ROS-mediated transformation from prostatitis to malignancy. Furthermore, spatially resolved transcriptomic techniques, integrated with single-cell analysis and proteomics, facilitate the construction of spatiotemporal maps of ROS-related molecules. The application of advanced research tools will further provide strong technical support and a solid theoretical foundation for the clinical translation of ROS-related research (**Figure 3**).

## 8 Conclusion

This article focuses on the role of ROS in the malignant transformation from prostatitis to PCa and explores its potential applications. As a crucial signaling molecule in oxidative stress, ROS is intricately linked to the progression of prostate tissue from inflammation to cancer. It can promote the progression of prostate diseases through multiple mechanisms, including the induction of DNA damage, activation of inflammatory signaling pathways, disruption of the balance between cell proliferation and apoptosis, and regulation of the HIF-1α-mediated hypoxia response and the NF-κB-driven inflammation-tumor signaling axis. In-depth research on the characteristics and functions of ROS is anticipated to yield novel ideas and strategies for the prevention and treatment of prostate diseases. However, significant uncertainties persist regarding the specific mechanisms of action, applicable scope, and potential risks associated with ROS in the onset and progression of PCa. Further basic research and clinical trials are urgently needed to enhance the understanding and application of its potential in treating prostate diseases. Future research should also emphasize the clinical translation and application of ROS, actively exploring combined treatment models that integrate ROS-related therapeutic strategies with other therapies. In conclusion, as a core regulatory molecule that connects oxidative stress, inflammatory responses, and tumor metabolism, the research on the mechanisms of ROS holds significant scientific value and potential clinical applications. However, further in-depth studies are necessary to elucidate its precise mechanisms of action in prostate diseases and to develop appropriate intervention strategies accordingly.
